# Bioprospecting of novel thermostable β-glucosidase from *Bacillus subtilis* RA10 and its application in biomass hydrolysis

**DOI:** 10.1186/s13068-017-0932-8

**Published:** 2017-10-30

**Authors:** Rameshwar Tiwari, Puneet Kumar Singh, Surender Singh, Pawan K. S. Nain, Lata Nain, Pratyoosh Shukla

**Affiliations:** 1 Enzyme Technology and Protein Bioinformatics Laboratory, Department of Microbiology, Maharshi Dayanand University, Rohtak, Haryana 124001 India; 2 Division of Microbiology, Indian Agricultural Research Institute, New Delhi, 110012 India; 3 Indian Institute of Technology Delhi, Hauz Khas, New Delhi, 110 016 India; 4 Design and Mechatronic Division, School of Civil and Mechanical Engineering, Galgotias University, Noida, Uttar Pradesh 201312 India

**Keywords:** Thermotolerant, Thermostable β-glucosidase, Saccharification, Cloning and expression, Molecular modelling

## Abstract

**Background:**

Saccharification is the most crucial and cost-intensive process in second generation biofuel production. The deficiency of β-glucosidase in commercial enzyme leads to incomplete biomass hydrolysis. The decomposition of biomass at high temperature environments leads us to isolate thermotolerant microbes with β-glucosidase production potential.

**Results:**

A total of 11 isolates were obtained from compost and cow dung samples that were able to grow at 50 °C. On the basis of qualitative and quantitative estimation of β-glucosidase enzyme production, *Bacillus subtilis* RA10 was selected for further studies. The medium components and growth conditions were optimized and β-glucosidase enzyme production was enhanced up to 19.8-fold. The β-glucosidase from *B. subtilis* RA10 retained 78% of activity at 80 °C temperature and 68.32% of enzyme activity was stable even at 50 °C after 48 h of incubation. The supplementation of β-glucosidase from *B. subtilis* RA10 into commercial cellulase enzyme resulted in 1.34-fold higher glucose release. Furthermore, β-glucosidase was also functionally elucidated by cloning and overexpression of full length GH1 family β-glucosidase gene from *B. subtilis* RA10. The purified protein was characterized as thermostable β-glucosidase enzyme.

**Conclusions:**

The thermostable β-glucosidase enzyme from *B. subtilis* RA10 would facilitate efficient saccharification of cellulosic biomass into fermentable sugar. Consequently, after saccharification, thermostable β-glucosidase enzyme would be recovered and reused to reduce the cost of overall bioethanol production process.

**Electronic supplementary material:**

The online version of this article (doi:10.1186/s13068-017-0932-8) contains supplementary material, which is available to authorized users.

## Background

Second generation biofuel production has been proposed as a sustainable and alternative energy option to sustain global energy demand [1]. Since, cellulose is the most abundant renewable biopolymer produced photosynthetically, the ultimate aim is to develop an economic industrially feasible process for conversion of lignocellulosic biomass into biofuel molecules [2]. The efficient hydrolysis of cellulose is most important step to develop biobased refineries. The saccharification step needs cellulases which have higher hydrolytic efficiency with increased stability at diverse pH, temperature and tolerance to end product inhibition [3, 4]. Among the cellulase enzyme complex, β-glucosidase is the rate-limiting enzyme and most of the fungal enzyme preparation do not produce sufficient β-glucosidase enzyme for complete conversion of cellulose into monomeric sugars [5]. Therefore, the main aim of the study to isolate novel β-glucosidase producers from unique habitats.

β-Glucosidases are widely distributed in the living world and they play pivotal roles in many biological processes. The physiological roles associated with this enzyme are diverse and depend on the location of the enzyme and the biological system in which these occur. In cellulolytic microorganisms, β-glucosidase is involved in cellulase induction and cellulose hydrolysis. In plants, the enzyme is involved in β-glucan synthesis during cell wall development, pigment metabolism, fruit ripening and defense mechanisms, whereas in humans and other mammals, β-glucosidases are thought to play a role in metabolism of glycolipids and dietary glucosides. Due to their wide and varied roles in nature, these versatile enzymes can be used in several industrial applications.

The microbial community can exist in the most diverse and extreme condition of this earth [6]. These ‘extremophiles’ are known to produce biocatalysts with special functional adaptation [7]. Consequently, the environmental samples from different niches may be explored for the novel biocatalysts with the unique characteristics which may find application in industries [8, 9]. Although there are several reports available on microbial community analysis from diverse extreme environments, the microbial community of herbivorous intestine is less explored which may be the main source of diverse hydrolytic enzymes involved in degradation of cellulose [10, 11]. Secondly, compost obtained from agricultural residues is also a rich habitat for lignocellulolytic microorganism, as it contains all organic substrates necessary for the growth of diverse microbial population [12, 13]. Therefore, both the niches were investigated for isolation of thermotolerant microorganisms with high β-glucosidase enzyme activities.

Selection for the potential β-glucosidase enzymes with enhanced activities remains a confront for the biorefinery industry to obtain efficient enzymatic hydrolysis of biomass [14]. With the advancement of recombinant DNA technology (RDT), now it is possible to overexpress the enzyme of interest in high titer with the ease of downstream processing for product development [15]. This technique has been successfully opted to produce industrially important biocatalysts and also open new avenues to understand the structure–function relationship. In this study, RDT was adapted for heterologous overexpression of glycosyl hydrolase family 1 β-glucosidase (*Bgl*B) from *B. subtilis* RA10.

## Methods

### Sample collection, enrichment and isolation of cellulolytic microorganisms

Compost (CM) prepared from paddy straw as raw material was collected from the compost pits of Division of Microbiology, IARI, New Delhi, India, in the month of September (2013). At the time of samples collection, compost heap was in the thermophilic phase with temperature ranging between 60 and 65 °C. Cow dung (CD) was collected from dairy farm situated in Todapur village, New Delhi, India. For isolation of cellulolytic microorganisms, the samples were enriched with α-cellulose or cellobiose. Samples (5 g) were incubated in 100 mL Erlenmeyer flask with 50 mL of Reese’s minimal medium (RMM) with 1% cellobiose. Enrichment flasks were incubated at 45 °C for 10 days. The diluted enriched medium was spread plated over the solid Reese’s minimal medium containing respective substrate and plates were incubated at 45 °C for 7 days.

### Qualitative screening of isolates for β-glucosidase production ability

Initially, selected morphotypes were qualitatively screened for β-glucosidase activity by testing them on RMM agar medium containing 0.5% esculin and 0.1% ammonium iron (III) citrate [11]. Isolates were grown overnight in nutrient broth at 45 °C and further cultures were spotted on esculin plates and incubated at 45 °C for 48 h. The β-glucosidase producing isolates were selected based on appearance of black zone around the colonies indicating the hydrolysis of esculin.

### Quantitative screening of isolates during growth on different carbon sources under submerged fermentation

The selected isolates based on esculin hydrolysis were screened for the production of β-glucosidase enzyme using different cellulosic carbon sources (Avicel PH101, Sigmacell 101, α-cellulose and cellobiose). This quantitative screening was performed under submerged fermentation using RMM containing 1% of these cellulosic substrates. Overnight grown culture was used as inoculum and flasks were incubated at 45 °C for 48 h in incubator shaker (150 rpm). All experiments were performed in triplicate. After growth, the medium was centrifuged at 10,000*g* for 10 min at 4 °C for the collection of crude enzyme.

### Quantitative assay of extracellular hydrolytic enzymes

The crude enzyme was assayed for hydrolytic enzymes based on the standard methods. The β-glucosidase (cellobiase; EC: 3.2.1.21) and β-xylosidase (EC: 3.2.1.37) activities were measured using 5 mM of *p*-nitrophenyl-β-d-glucopyranoside (pNPG) and *p*-nitrophenyl-β-d-xylopyranoside (pNPX) as a substrate, respectively. The quantitative assay of other hydrolytic enzymes, e.g. Filter paperase (FPase), endoglucanase (CMCase; EC: 3.2.1.4) and xylanase (EC: 3.2.1.8) was performed spectrophotometrically by measuring the reducing sugar released by culture filtrate as a result of hydrolysis of Whatman No. 1 filter paper (50 mg), carboxymethyl cellulose (CMC) (2%, w/v) and birch wood xylan (1%, w/v), respectively [16, 17]. The reducing sugar was estimated by DNSA method [18]. One unit of enzyme represents 1 μM of end product released mL^−1^ min^−1^.

### Molecular identification and phylogenetic analysis of selected strains

All isolates were identified using PCR amplification of 16S ribosomal gene sequence based on molecular characterization technique. The overnight grown cultures were pelleted out and genomic DNA was isolated by using Zymo Research Fungal/Bacterial DNA MicroPrep™ kit. Partial 16S rRNA gene fragment of approximately 1.5 kb was amplified using universal PA (5′ AGA GTT TGA TCC TGG CTC AG 3′) and PH (5′ AAG GAG GTG ATC CAG CCG CA 3′) primer set. The amplified PCR products were outsourced to Eurofins Genomics India Pvt Ltd., Bangalore, India, for DNA sequencing. The 16S rRNA partial gene sequences of the isolates were compared by the NCBI-BLAST search (http://www.ncbi.nlm.nih.gov) to identify the nearest taxa. The 16S rRNA gene sequences were aligned for the construction of phylogenetic dendrogram. The tree topologies were calculated by bootstrap analysis of 1000 datasets using MEGA 5.0 software. The sequences were also submitted to NCBI Genbank.

### Selection of media components by using Taguchi experimental design

The effect of different media components on β-glucosidase enzyme production by *Bacillus subtilis* RA10 was deduced by Taguchi optimization. The selected factors with their respective levels used for experiment are listed in Table 1. The design of experiment suggested a L_25_(5^5^) orthogonal array for the optimization, and totally 25 experiments were performed in triplicate with different combinations of five factors and respective five levels (Table 2). After experimentation, the data were analysed by analysis of variance (ANOVA) using Minitab^®^ 16.2.1 software [19].

**Table 1:** Factors and their levels employed in the Taguchi experimental design

S. no.	Factor	Units	Level 1	Level 2	Level 3	Level 4	Level 5
1	Carbon source	(1%, w/v)	Paddy straw	Wheat bran	Baggase	Wheat straw	*Parthenium*
2	Organic N source	(0.1%, w/v)	Yeast extract	Malt extract	Beef extract	Peptone	Tryptone
3	pH	–	6	7	8	9	10
4	Surfactant	(0.1%, v/v)	Tween 20	Tween 80	Triton X 100	PEG 6000	–
5	Inorganic N source	(0.1%, w/v)	NH_4_Cl	NaNO_3_	(NH_4_)_2_HPO_4_	NH_4_NO_3_	(NH_4_)_2_SO_4_

**Table 2:** Design and experimental results of the L_25_(5^5^) orthogonal array of Taguchi experimental design for β-glucosidase activity from *B. subtilis* RA10

S. no.	Carbon source	Organic N source	pH	Surfactant	Inorganic N source	β-Glucosidase activity (IU/g)
1	Paddy straw	Yeast extract	6	Tween 20	NH_4_Cl	25.00
2	Paddy straw	Malt extract	7	Tween 80	NaNO_3_	32.37
3	Paddy straw	Beef extract	8	Triton X 100	(NH_4_)_2_HPO_4_	31.85
4	Paddy straw	Peptone	9	PEG 6000	NH_4_NO_3_	32.77
5	Paddy straw	Tryptone	10	–	(NH_4_)_2_SO_4_	21.32
6	Wheat bran	Yeast extract	8	Tween 80	NH_4_NO_3_	25.74
7	Wheat bran	Malt extract	9	Triton X 100	(NH_4_)_2_SO_4_	22.26
8	Wheat bran	Beef extract	10	PEG 6000	NH_4_Cl	19.37
9	Wheat bran	Peptone	6	–	NaNO_3_	17.4
10	Wheat bran	Tryptone	7	Tween 20	(NH_4_)_2_HPO_4_	21.32
11	Baggase	Yeast extract	10	Triton X 100	NaNO_3_	26.47
12	Baggase	Malt extract	6	PEG 6000	(NH_4_)_2_HPO_4_	17.12
13	Baggase	Beef extract	7	–	NH_4_NO_3_	15.95
14	Baggase	Peptone	8	Tween 20	(NH_4_)_2_SO_4_	20.06
15	Baggase	Tryptone	9	Tween 80	NH_4_Cl	15.22
16	Wheat straw	Yeast extract	7	PEG 6000	(NH_4_)_2_SO_4_	22.38
17	Wheat straw	Malt extract	8	–	NH_4_Cl	16.76
18	Wheat straw	Beef extract	9	Tween 20	NaNO_3_	33.95
19	Wheat straw	Peptone	10	Tween 80	(NH_4_)_2_HPO_4_	27.02
20	Wheat straw	Tryptone	6	Triton X 100	NH_4_NO_3_	21.82
21	*Parthenium*	Yeast extract	9	–	(NH_4_)_2_HPO_4_	11.03
22	*Parthenium*	Malt extract	10	Tween 20	NH_4_NO_3_	17.32
23	*Parthenium*	Beef extract	6	Tween 80	(NH_4_)_2_SO_4_	16.32
24	*Parthenium*	Peptone	7	Triton X 100	NH_4_Cl	19.63
25	*Parthenium*	Tryptone	8	PEG 6000	NaNO_3_	15.82

### Optimization of selected media components by using Box–Behnken design

After the Taguchi-based optimization, Box–Behnken statistical method was chosen to optimize the concentrations of selected medium components. Therefore, medium components were chosen for optimizing their concentration for β-glucosidase enzyme production using *B. subtilis* RA10 (Table 3). The Design-Expert^®^ Software was used for experiment design and analysis. A total of 27 experiments were conducted based on the design matrix with three centre points to minimize the experimental error (Table 4).

**Table 3:** Experimental range and coded levels employed in the response surface methodology (RSM) using the Box–Behnken design for increasing the production of β-glucosidase enzymes by *B. subtilis* RA10

	Process variable	Range and level		
		− 1	0	+ 1
*A*	Paddy straw (%, w/v)	1	2	3
*B*	Beef extract (%, w/v)	0.1	0.25	0.4
*C*	PEG 6000 (%, v/v)	0.1	0.25	0.4
*D*	NaNO_3_ (%, w/v)	0.1	0.25	0.4

**Table 4:** Box–Behnken design matrix for the experimental design and respective response for β-glucosidase activity from *B. subtilis* RA10

Std. order	Experimental coded values of different variables				β-Glucosidase activity (IU/g)
	Paddy straw	Beef extract	PEG 6000	NaNO_3_	Actual
1	1	0.1	0.25	0.25	37.67
2	3	0.1	0.25	0.25	47.12
3	1	0.4	0.25	0.25	30.41
4	3	0.4	0.25	0.25	33.15
5	2	0.25	0.1	0.1	36.48
6	2	0.25	0.4	0.1	34.42
7	2	0.25	0.1	0.4	42.97
8	2	0.25	0.4	0.4	44.52
9	1	0.25	0.25	0.1	36.85
10	3	0.25	0.25	0.1	42.95
11	1	0.25	0.25	0.4	46.64
12	3	0.25	0.25	0.4	54.74
13	2	0.1	0.1	0.25	37.33
14	2	0.4	0.1	0.25	26.71
15	2	0.1	0.4	0.25	35.57
16	2	0.4	0.4	0.25	24.95
17	1	0.25	0.1	0.25	38.72
18	3	0.25	0.1	0.25	34.15
19	1	0.25	0.4	0.25	27.29
20	3	0.25	0.4	0.25	43.05
21	2	0.1	0.25	0.1	41.42
22	2	0.4	0.25	0.1	28.55
23	2	0.1	0.25	0.4	48.96
24	2	0.4	0.25	0.4	40.60
25	2	0.25	0.25	0.25	37.03
26	2	0.25	0.25	0.25	37.03
27	2	0.25	0.25	0.25	37.03

### Optimization of incubation temperature and time for β-glucosidase enzyme production

The optimization of incubation temperature and time was performed for higher production of β-glucosidase enzyme from *B. subtilis* RA10. The enzyme was produced under optimized condition and extracted periodically up to 144 h. The β-glucosidase production was evaluated at different temperatures within 5–60 °C.

### β-Glucosidase production in bioreactor

For large-scale production of β-glucosidase, *B. subtilis* RA10 was grown under optimized conditions in 7 L stirred tank bioreactor (Applikon, Schiedam, Netherlands) controlled by Applikon Bio Controller, Bio Console ADI1025. The bioreactor experiment was performed with automatic temperature control at 40 °C, 8.0 pH and 200 rpm of agitation rate. The dissolved oxygen was maintained at 70% by regulating the aeration of 1 L/min with working volume of 5 L. The pH was controlled by the automatic addition of either 5% (v/v) H_2_SO_4_ or 5% (v/v) NaOH. Overnight grown culture (10^5^ CFU/mL) of *B. subtilis* RA10 in nutrient broth was used as primary inoculum. Samples were drawn periodically up to 96 h. The crude enzyme was centrifuged at 10,000*g* for 10 min and used for the assay of hydrolytic enzymes and protein content.

### Enzymatic saccharification of pretreated paddy straw

After optimization and characterization of β-glucosidase enzyme from *B. subtilis* RA10, the acetone-precipitated crude extract was evaluated for the hydrolysis of pretreated paddy straw. The concentrate crude extract contained β-glucosidase (78 IU/mL), endoglucanase (0.23 IU/mL), FPase (0.8 IU/mL) and xylanase (4.2 IU/mL) activities. Paddy straw was pretreated with sodium hydroxide as described by Tiwari et al. [20]. The composition of alkali pretreated paddy straw on a dry weight basis was 562 mg/gds of cellulose, 168 mg/gds of xylan and 81 mg/gds of lignin. Cellulase from *Trichoderma reesei* (Celluclast^®^ 1.5L) was used as commercial cellulase enzyme preparation with FPase (98.05 IU/mL), CMCase (137.7 IU/mL), β-glucosidase (54.83 IU/mL) and xylanase (152.5 IU/mL). Enzymatic hydrolysis of alkali pretreated paddy straw was performed in four sets. Firstly, commercial cellulase (Celluclast^®^ 1.5L) (30 FPU/gds) and crude enzyme prepared from *B. subtilis* RA10 (15 β-glucosidase units/gds correspond to 0.15 FPU/gds) were used separately for biomass hydrolysis. In another set, Celluclast^®^ 1.5L was supplemented with crude enzyme extract as a source of β-glucosidase produced by *B. subtilis* RA10. The fourth set of experiment was conducted to evaluate the effectiveness of FPU present in crude extract of *B. subtilis*. Thus, Celluclast^®^ 1.5L enzyme with 30.15 FPU/gds was used for biomass hydrolysis. The saccharification of pretreated paddy straw was carried out in 50 mM citrate buffer (pH 4.8) with 0.01% sodium azide at 5% substrate loading (dry weight basis). Experiments were performed at 50 °C under shaking condition (150 rpm) for 72 h. Samples were withdrawn periodically and analysed for sugar concentration by high-performance liquid chromatography (Waters pump 515 model) equipped with Waters 2414 refractive index (RI) detector.

### Amplification of full length β-glucosidase gene

The genomic DNA isolated from *B. subtilis* RA10 was used as a template to amplify β-glucosidase gene. A total of 100 β-glucosidase protein sequences were retrieved from NCBI Genbank by selecting Firmicutes as organism group. All sequences were aligned using T-Coffee alignment (http://www.tcoffee.org/Projects/tcoffee). The degenerate primer set was designed for conserved amino acid sequence position 1–7 (MIHKKQG) and 473 to 478 (TNGKSL). The complete gene of β-glucosidase was amplified by degenerate primer set BGF (5′ GGC C**GG ATC C**AT GAT HCA YAA RAA RCA RGG I 3′) and BGR (5′ GGC C**GT CGA C**IA RIS WYT TIC CRT TIG T 3′) having *Bam*HI restriction site (bold in forward primer) and *Sal*I restriction site (bold in reverse primer). The PCR amplification was conducted with Pfu DNA polymerase for high fidelity.

### Cloning and expression of β-glucosidase gene

Amplified PCR product was cloned into pGEM-T easy vector (Promega, Wallisellen, Switzerland). The β-glucosidase gene was extracted from the positive clone with M13F and M13R primers and delivered for sequencing for conformation. β-glucosidase genes cloned into pGEM-T easy vector and pET28a^+^ (Novagen) were digested with *Bam*HI and *Sal*I restriction enzymes and run along with marker on 1% agarose gel. The ligated product was transformed into *E. coli* strain BL21 (DE3) competent cells.

### Purification of recombinant protein

The transformant carrying the *Bgl*B + pET28a^+^ plasmid was grown up to log phase and expressed the recombinant protein as described previously. The cells were harvested by centrifugation at 6500 rpm for 10 min and resuspended in buffer A (50 mM Tris- HCl and 200 mM NaCl, pH 7.5). The cell suspension was sonicated (6 × 10 s, Sonicator Heat system with 50% duty cycle) on ice and clear supernatant was collected for further purification. The overexpressed recombinant protein was purified by using electroelution technique. The soluble fraction of protein was run on two parallel native gels and after the completion of gel run, one gel was stained with Coomassie brilliant blue R-250 to visualize the overexpression. Thereafter, based on the location of overexpressed band, unstained gel was cut at the same site using a surgical blade. The gel slice was again chopped and transferred into the dialysis bag containing sodium citrate buffer (0.05 M, pH 4.8). The dialysis bag was kept horizontally on agarose gel electrophoresis unit filled with sodium citrate buffer (0.05 M, pH 4.8) and operated for 50 V for 1 h. After the electrophoresis, the extract was analysed through 12% SDS-PAGE as described previously and stained with Coomassie brilliant blue R-250. The extract was further utilized for the characterization purpose.

### Characterization of purified recombinant protein

The reaction velocity of the purified enzyme was determined using 0.5–5.0 mM *p*-nitrophenyl-β-d-glucopyranoside in citrate buffer (50 mM) of pH 5.0. The kinetic constants *K*_m_ and *k*_cat_ were calculated by a nonlinear regression of Michaelis–Menten equation using GraphPad PRISM version 6.05 (trial version). The purified enzyme was further investigated to study the effect of temperature on β-glucosidase enzyme between 10 and 80 °C. The optimal pH was analysed by estimating the activities between pH 3.0 and 10.0, using 50 mM citrate (pH 3.0–6.0), sodium phosphate (pH 6.0–9.0) and glycine–NaOH (pH 9.0–10.0) buffers. The inhibitory effect of glucose on β-glucosidase activity was measured by adding different d-glucose concentrations (0–1000 mM). For the determination of temperature and pH stability, the enzyme was pre-incubated in the temperature range of 20, 30, 40, 50, 60 and 80 °C for 0–16 h, or in the initial pH range 3–7 at 30 °C, and assayed for β-glucosidase activity.

### Sequence alignment, modelling and docking studies for full length amino acid sequence of BglB

Full-length amino acid sequence of *Bgl*B was aligned with the other GH1 family β-glucosidase protein sequences by ClustalW. The homology model was also built using the automated SWISS-MODEL server, using β-glucosidase (PDB code 2XHY_A) as a template. The modelled structures were further verified by PROCHEK and Verify 3D. The docking was carried out with AutoDock4 (http://autodock.scripps.edu).

## Results

### Isolation and identification of thermotolerant microorganisms

**Fig. 1:**
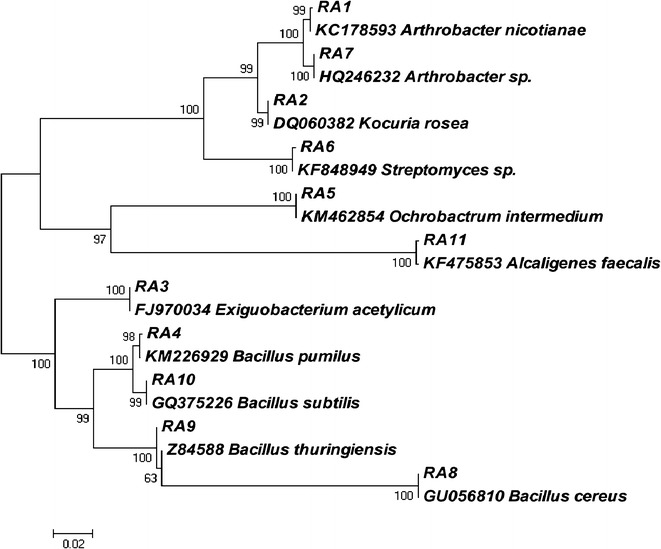
Phylogenetic trees based on ribosomal gene sequences showing the relationship of all strains obtained from compost and cow dung with their nearest phylogenetic relatives

The total viable count (TVC) of thermotolerant microbes ranged from 10^3^ to 10^5^ CFU (colony forming unit)/g of sample. A total of 6 and 5 morphotypes were selected based on the colony morphology from compost and cow dung samples, respectively. Their capability to produce β-glucosidase enzyme was confirmed on the basis of the formation of dark brown zone on esculin plate at 40 °C (Additional file 1: Figure S1). For the identification of these bacterial isolates, the 16S rRNA gene was successfully amplified (about 1500 bp) from the eleven isolates. The isolates comprised seven different genera including *Bacillus* (*Bacillus pumilus* RA4, *Bacillus cereus* RA8, *Bacillus thuringiensis* RA9, *Bacillus subtilis* RA10), *Arthrobacter* (*Arthrobacter nicotianae* RA1, *Arthrobacter* sp. RA7), *Exiguobacterium* (*Exiguobacterium acetylicum* RA3), *Kocuria* (*Kocuria rosea* RA2), *Ochrobactrum* (*Ochrobactrum intermedium* RA5), *Streptomyces* (*Streptomyces* sp. RA6) and *Alcaligenes* (*Alcaligenes faecalis* RA11) (Table 5). All these isolates lie in four different phyla including Actinobacteria, Firmicute, α-Proteobacteria and β-Proteobacteria. The phylogenetic relationship of each thermotolerant isolate with their respective nearest neighbour is presented in Fig. 1.

**Table 5:** Description and phylogenetic affiliation of selected isolates from compost (CM) and cow dung (CD)

S. no.	Isolate	Sample type	Gram stain	Colony colour	Division	Most similar neighbour	Identity (%)	GenBank accession number
1	RA1	CM	Positive	White	Actinomycetales	*Arthrobacter nicotianae*	98	KT898113
2	RA2	CM	Positive	White	Actinomycetales	*Kocuria rosea*	99	KT898114
3	RA3	CD	Positive	Orange	Firmicute	*Exiguobacterium acetylicum*	99	KT898115
4	RA4	CD	Positive	White	Firmicute	*Bacillus pumilus*	98	KT898116
5	RA5	CD	Negative	Cream	α-Proteobacteria	*Ochrobactrum intermedium*	99	KT898117
6	RA6	CD	Positive	White	Actinomycetales	*Streptomyces* sp.	99	KT898118
7	RA7	CM	Positive	White	Actinomycetales	*Arthrobacter* sp.	99	KT898119
8	RA8	CM	Positive	White	Firmicute	*Bacillus cereus*	98	KT898120
9	RA9	CD	Positive	White	Firmicute	*Bacillus thuringiensis*	99	KT898121
10	RA10	CM	Positive	White	Firmicute	*Bacillus subtilis*	99	KT898122
11	RA11	CM	Negative	White	β-Proteobacteria	*Alcaligenes faecalis*	98	KT898123

### Evaluation of selected isolates for β-glucosidase enzyme production using saccharides as carbon source

**Fig. 2:**
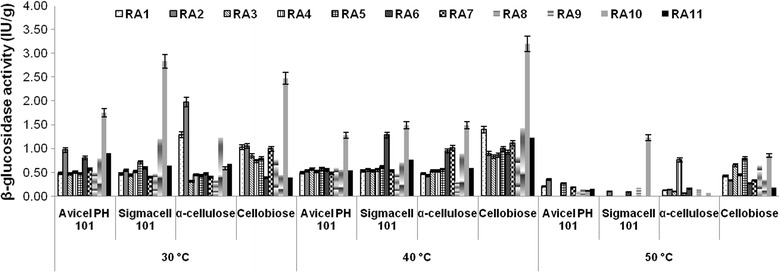
Effect of different carbon sources on the production of β-glucosidase by selected isolates from compost and cow dung at different temperatures

All the thermotolerant isolates obtained from compost and cow dung were able to grow and produce β-glucosidase enzyme at 30, 40 and 50 °C. Of all isolates, *B. subtilis* RA10 produced maximum β-glucosidase enzyme (3.19 ± 0.13 IU/g) at 40 °C by using cellobiose as carbon source (Fig. 2) which was selected for further optimization studies.

### Effect of different media components on β-glucosidase enzyme production by *B. subtilis* RA10

β-Glucosidase enzyme production by thermotolerant *B. subtilis* RA10 was optimized by Taguchi design. The experimental run gave β-glucosidase enzyme production ranging from 11.03 to 33.95 IU/g (Table 2). The ANOVA table indicated that all the factors play a significant (*P* < 0.05) role in the enzyme production. The significance of model was revealed by 97% of coefficient of variation (*R* ^2^) (Table 6). On the basis of contribution percentage, carbon source was examined as the most influential factor with 52.12% contribution followed by surfactant (22.05%) and inorganic N source (11.45%). Other factors also showed significant effect on enzyme production. The validation experiment confirms that paddy straw as carbon source, beef extract and NaNO_3_ as organic and inorganic N source, PEG 6000 as surfactant and pH 8 were the optimum medium component for maximizing the β-glucosidase enzyme production (36.64 ± 0.28 IU/g) by *B. subtilis* RA10. After optimization by using Taguchi design, the β-glucosidase enzyme production was 11.5-fold higher as compared with previous experiment under unoptimized condition.

**Table 6:** Analysis of variance (ANOVA) of β-glucosidase enzyme production by *B. subtilis* RA10 by using Minitab 16 software based on L_25_(5^5^) standard orthogonal array experiment

S. no.	Factors	DOF	Sum of squares	Variance	*F* value	*P* value	Contribution (%)	Rank
1	Carbon source	4	477.527	119.382	16.04	0.00	52.12	1
2	Organic N source	4	65.586	16.396	2.2	0.00	7.16	4
3	Surfactant	4	201.971	50.493	6.78	0.00	22.05	2
4	pH	4	36.374	9.094	1.22	0.00	3.97	5
5	Inorganic N source	4	104.898	26.224	3.52	0.00	11.45	3
	Error	54	29.78	7.445			3.25	
	Total	74	916.135				100.00	

*S* = 2.72854, *R* ^2^ = 0.97, *R* ^2^(adj) = 0.93

### Optimization of the concentration of selected medium component using RSM

β-Glucosidase enzyme production by *B. subtilis* RA10 was optimized by Box–Behnken design to evaluate the interaction between different independent variables and their levels. Based on Taguchi optimization, four selected medium components, namely, (*A*) paddy straw, (*B*) beef extract, (*C*) PEG 6000 and (*D*) NaNO_3_ were further investigated for their optimum combination using RSM following Box–Behnken design matrix. The experimental data of β-glucosidase enzyme production by *B. subtilis* RA10 are mentioned in Table 4. Four selected factors includes Paddy straw (1–3% (w/v)), Beef extract (0.1–0.4% (w/v)), PEG 6000 (0.1–0.4% (w/v)) and NaNO_3_ (0.1–0.4% (w/v)). The results obtained after Box–Behnken design were subjected to ANOVA (Table 7) and fitted with the polynomial equation. Based on quadratic model of response, the equation in terms of coded variable and actual variables was as follows:

$$\begin{aligned} \upbeta{\text{-Glucosidase }} &= + 37.03 + 3.13 * A - 5.31 * B - 0.55 * C \\ & \quad + 4.81 * D {-} 1.68 * A * B + 5.08 * A * C \\ & \quad + 0.50 * A * D + 0.00 * B * C + 1.13 * B * D \\ & \quad + 0.90 * C * D + 2.44 * A^{2} - 2.59 * B^{2} \\ & \quad - 3.38 * C^{2} + 5.74 * D^{2}\end{aligned}.$$

**Table 7:** Regression analysis for the production of β-glucosidase enzyme from *B. subtilis* RA10 for quadratic response surface model fitting (ANOVA)

Source	Sum of squares	*df*	Mean square	*F* value	*P* value Prob > *F*	
Model	1311.03	14	93.64	170.69	< 0.0001	Significant
*A* paddy straw	117.65	1	117.65	214.45	< 0.0001	
*B* beef extract	338.07	1	338.07	616.22	< 0.0001	
*C* PEG 6000	3.57	1	3.57	6.507	0.0254	
*D* NaNO_3_	277.96	1	277.96	506.65	< 0.0001	
*AB*	11.24	1	11.24	20.48	0.0007	
*AC*	103.34	1	103.34	188.36	< 0.0001	
*AD*	1.00	1	1.00	1.82	0.2019	
*BC*	0.00	1	0.00	0	1.0000	
*BD*	5.10	1	5.10	9.28	0.0101	
*CD*	3.25	1	3.25	5.92	0.0315	
*A* ^2^	31.86	1	31.86	58.06	< 0.0001	
*B* ^2^	35.85	1	35.85	65.34	< 0.0001	
*C* ^2^	60.89	1	60.89	110.99	< 0.0001	
*D* ^2^	175.65	1	175.65	320.17	< 0.0001	
Residual	6.58	12	0.548615			
Lack of fit	6.58	10	0.658337		0.08	Not significant
Pure error	0.00	2	0			

**Fig. 3:**
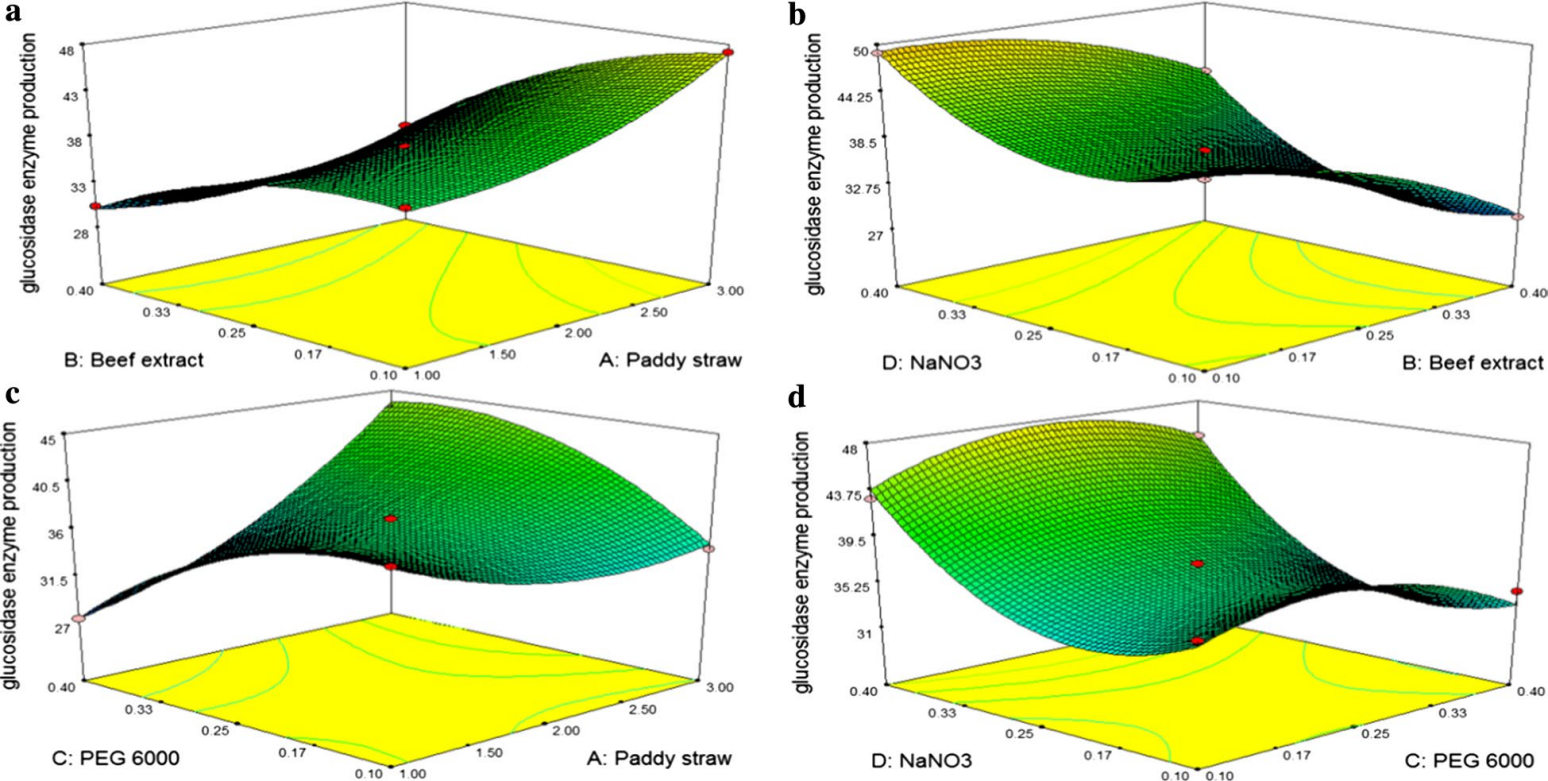
Response surface plots of Box–Behnken design for optimization of the β-glucosidase enzyme production by *B. subtilis* RA10. Figure shows the interaction between **a** beef extract and Paddy straw, **b** NaNO_3_ and beef extract, **c** PEG 6000 and paddy straw, **d** NaNO_3_ and PEG 6000

The quadratic type model was produced and all the effects were found to be significant (*P* < 0.05). Among six interactions, two [paddy straw/NaNO_3_ (*AD*) and Beef extract/PEG 6000 (*BC*)] displayed insignificant *P* value (*P* > 0.05) and therefore suggested insignificant interaction effect on β-glucosidase production. The rest of the four interactions between beef extract/paddy straw, NaNO_3_/beef extract, PEG 6000/paddy straw and NaNO_3_/PEG 6000 displayed that their effect on β-glucosidase production was significant but not linear. The interactive effect of factors on β-glucosidase production was also analysed by 3D contour plots against two experimental factors (Fig. 3).

The developed model was validated by performing the experiment under predicted optimal condition for maximizing the β-glucosidase enzyme production by *B. subtilis* RA10. The model proposed paddy straw—1.24% (w/v), beef extract—0.29% (w/v), PEG 6000—0.14% (v/v) and NaNO_3_—0.12% (w/v) as the optimum conditions with predicted β-glucosidase yield of 55.21 IU/g. The experimental β-glucosidase activity 57.31 ± 0.81 IU/g under optimized condition was in close agreement with the predicated β-glucosidase activity. After Box–Behnken design-based optimization, β-glucosidase enzyme production increased by 1.6-fold as compared to the result obtained after Taguchi optimization (36.64 ± 0.28 IU/g).

### Effect of incubation temperature and time on production of β-glucosidase enzyme by *B. subtilis* RA10

**Fig. 4:**
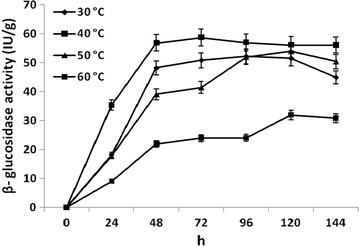
Effect of incubation temperature and time on the production of β-glucosidase enzyme from *B. subtilis* RA10 during submerged fermentation in flask

After the statistical optimization by Taguchi experiment and Box–Behnken design, the effect of incubation temperature and time on production of β-glucosidase enzyme was observed. *B. subtilis* RA10 produced β-glucosidase enzyme at broad range of incubation temperature ranging from 30 to 60 °C with the activity ranging between 31.94 ± 0.48 and 58.71 ± 1.73 IU/g (Fig. 4). The maximum β-glucosidase enzyme production (58.71 ± 1.73 IU/g) was achieved at 40 °C after 72 h of Incubation, and further incubation up to 144 h did not result in any increase in enzyme activity.

### β-Glucosidase production in bioreactor by *B. subtilis* RA10

**Fig. 5:**
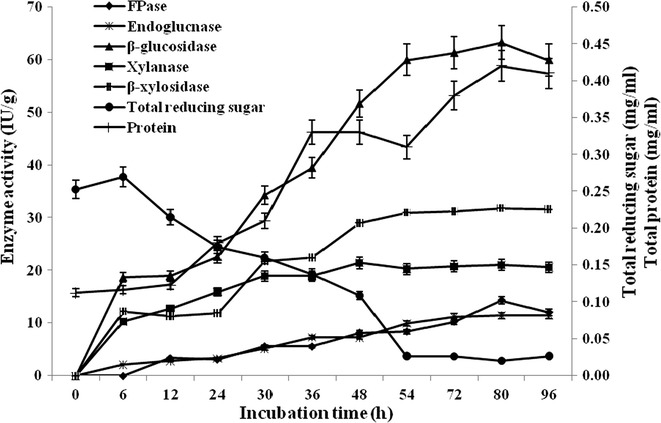
Profile of hydrolytic enzyme production by *B. subtilis* RA10 under optimized condition in 7 L fermentor

For large-scale β-glucosidase production, thermotolerant *B. subtilis* RA10 was grown under the optimized medium condition with 1.24% (w/v) of paddy straw, 0.29% (w/v) of beef extract, 0.14% (v/v) of PEG 6000 and NaNO_3_—0.12% (w/v) at 40 °C. The bacterial growth increased throughout the experiment and the population reached 12 × 10^8^ CFU/mL in liquid medium after 96 h of submerged fermentation. The extracellular protein also reached from 0.11 ± 0.03 to 0.42 ± 0.04 mg/mL. The maximum β-glucosidase yield of 63.29 ± 1.73 IU/g was achieved after 80 h of incubation which was 1.08-fold higher than the shake flask experiment. Thermotolerant *B. subtilis* RA10 also co-produced other hydrolytic enzymes like endoglucanase (11.04 ± 0.24 IU/g), FPase (12.00 ± 0.47 IU/g), xylanase (20.81 ± 0.38 IU/g) and β-xylosidase (31.32 ± 0.99 IU/g) after 96 h of incubation (Fig. 5).

### Saccharification of pretreated paddy straw by supplementing β-glucosidase enzyme from *B. subtilis* RA10

β-Glucosidase producing *B. subtilis* RA10 secretome was further evaluated for saccharification of alkali-pretreated paddy straw at 50 °C. This experiment was performed in three different sets: crude secretome of *B. subtilis* RA10; commercial cellulase (Celluclast^®^ 1.5L) and Celluclast^®^ 1.5L supplemented with crude secretome of *B. subtilis* RA10 as a source of indigenous β-glucosidase, for saccharification of alkali-pretreated paddy straw (Table 8). Initially, saccharification experiment was performed by crude secretome of *B. subtilis* RA10 alone, and small amount of glucose (45.34 ± 0.73 mg/gds) and xylose (8.84 ± 0.10 mg/gds) was released after 48 h of saccharification process. In another set, saccharification of pretreated paddy straw with commercial cellulase (Celluclast^®^ 1.5L) was performed and higher sugar release (437.79 ± 3.54 mg/gds) was observed. In third set of experiment, the supplementation of β-glucosidase with Celluclast^®^ 1.5L significantly improved the release of total sugars (582.58 ± 2.22 mg/gds) from the alkali pretreated paddy straw which was 1.33-fold higher than the sugar release obtained from the commercial cellulase alone. The effect of supplementation of β-glucosidase from *B. subtilis* RA10 into commercial Celluclast^®^ 1.5L was observed as the glucose release after the supplementation was 1.34-fold higher. In fourth set, 30.15 FPU/gds commercial cellulase (Celluclast^®^ 1.5L) was found to release 447.2 ± 1.94 mg/gds of total sugar which was comparable with second set of saccharification experiment.

**Table 8:** Sugar release after 72-h saccharification of alkali-pretreated paddy straw by cellulase from *T. reesei* ATCC 26921 (Celluclast^®^ 1.5L) supplemented with β-glucosidase from *B. subtilis* RA10

S. no.	Enzyme loading	Sugar release (mg/gds)		
		Glucose	Xylose	Total sugar
1	β-Glucosidase from *B. subtilis* RA10 (15 β-glucosidase unit/gds corresponds to 0.15 FPU/gds)	45.34 ± 0.73	8.84 ± 0.10	54.18 ± 0.83
2	Celluclast^®^ 1.5L (30 FPU/gds)	376.14 ± 2.36	61.65 ± 1.18	437.79 ± 3.54
3	Celluclast^®^ 1.5L (30 FPU/gds) supplemented with β-glucosidase (15 unit/gds) from *B. subtilis* RA10	506.16 ± 1.74	76.42 ± 0.48	582.58 ± 2.22
4	Celluclast^®^ 1.5L (30 + 0.15 = 30.15 FPU/gds)	384.38 ± 1.06	62.82 ± 0.88	447.2 ± 1.94

### PCR amplification, cloning and overexpression of BglB in *E. coli* BL21 (DE3)

The amplified product of full length *Bgl*B gene (1437 bp) was cloned in pGEM-T Easy vector and the ligated product was transformed into *E. coli* (DH5α) strain (Additional file 1: Figure S2). The positive white clone was selected and insert was validated by amplifying the insert by M13 primers. Upon digestion of the recombinant vector pGEM-T + *Bgl*B with *Bam*HI and *Sal*I restriction enzymes, the released fragments were obtained for further subcloning. For the expression of *Bgl*B in *E. coli*, the ORF was cloned in pET28a^+^ expression vector. The recombinant vector pGEM-T + *Bgl*B and pET28a^+^ vector was double digested with *Bam*HI and *Sal*I for directional cloning. The plasmid was isolated from these clones and digested with *Bam*HI and *Sal*I, which showed the presence of *Bgl*B gene. A positive clone was then chosen for expression studies.

**Fig. 6:**
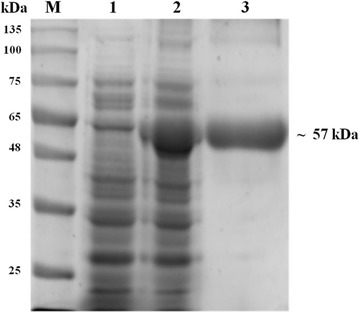
Heterologous expression and purification of β-glucosidase on 12% acrylamide gel and stained with Coomassie Blue. Lane M, molecular mass markers; Lane 1, cells carrying the vector with the insert before IPTG induction; Lane 2, total cellular protein after IPTG induction; Lane 3, purified recombinant β-glucosidase after electroelution

Cloned β-glucosidase gene *Bgl*B was overexpressed under the control of IPTG-inducible phage T7 promotor, which is predicted to encode a recombinant protein of 497 amino acids with a molecular weight of ~ 57.13 kDa. On analysis of protein on 12% SDS-PAGE, correct size recombinant protein band (~ 57.13 kDa) was observed. The results confirmed that the ORF was selectively expressed in the transformed *E. coli* BL21 (DE3) cells and apparently constituted a large fraction of the total protein. The overexpressed protein was purified by electroelution. The purified protein (~ 57.13 kDa) was also confirmed by SDS-PAGE (Fig. 6). The β-glucosidase enzyme activity was also measured in the soluble fraction and purified protein. The soluble fraction of the β-glucosidase enzyme activity was 7.83 ± 0.21 IU/mL with 13.50 ± 0.93 IU/mg of specific activity, while purified enzyme showed the enzyme activity of 5.27 ± 0.78 IU/mL with higher specific activity 47.91 ± 1.43 IU/mg.

### Kinetic parameters of BglB β-glucosidase enzymes

The purified protein obtained from previous experiment was also used to determine the enzyme kinetics by using *p*NPG as a substrate (Additional file 1: Figure S3). The *K*_m_ and *k*_cat_ of the β-glucosidase enzyme were found to be 3.90 ± 0.20 mM and 3.09 ± 0.02 s^−1^, respectively. The *V*_max_ was achieved as 12.05 ± 0.32 µM/min.

### Optimum temperature, pH and glucose for β-glucosidase enzyme

**Fig. 7:**
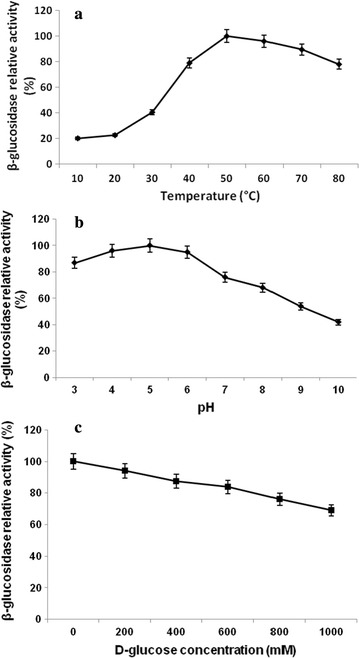
Effect of temperature (**a**), pH (**b**) and glucose concentration (**c**) on the purified β-glucosidase activity from *B. subtilis* RA10

**Fig. 8:**
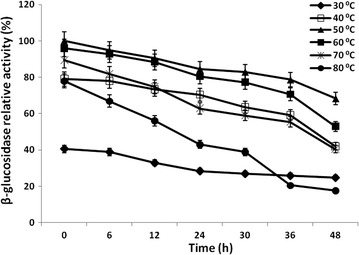
Temperature stability of β-glucosidase activity from *B. subtilis* RA10

To characterize the β-glucosidase enzyme for optimum pH and temperature, the activity of enzyme was measured at different temperatures (30–80 °C) and pH ranges (3.0–10.0). The purified enzyme retained 78% of β-glucosidase activity at 80 °C of temperature (Fig. 7a). The enzyme also retained 70% activity at alkaline pH 7.0 (Fig. 7b). The glucose inhibition studies showed very less effect of high glucose concentration on β-glucosidase activity. Purified β-glucosidase retained 70% of activity in the presence of 1000 mM of glucose concentration (Fig. 7c). β-glucosidase activity was checked for thermal stability at temperature range of 30–80 °C (Fig. 8). The enzyme remained active in a wide range of temperatures with highest activity (68.32%) at 50 °C after 48 h of incubation. However, β-glucosidase enzyme also retained 52.92, 40.42 and 17.55% of their activity at 60, 70 and 80 °C, respectively, after 48 h of incubation.

### Sequence alignment, modelling and docking studies

**Fig. 9:**
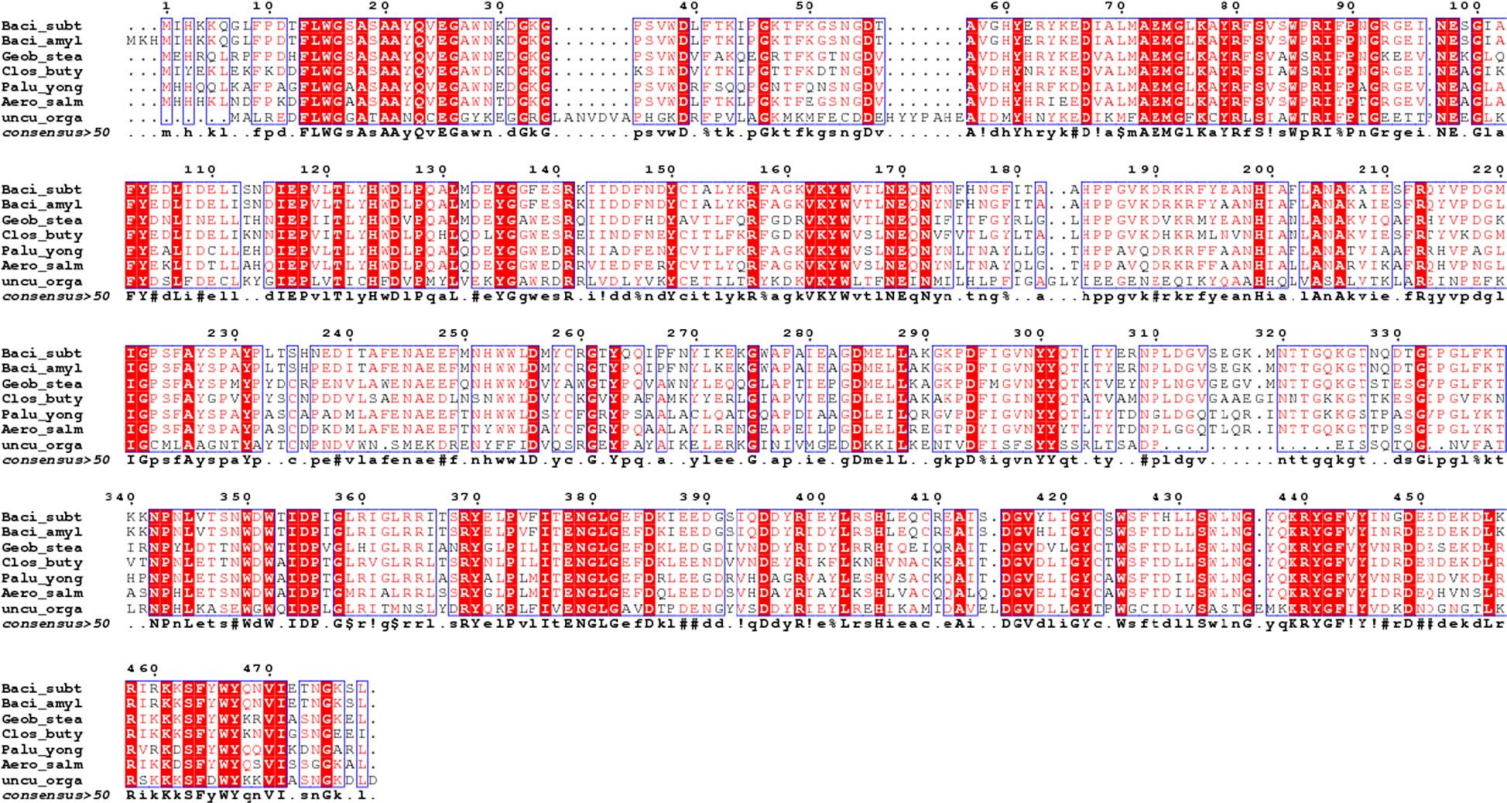
Multiple sequence alignment of BglB against the similar GH1 β-glucosidase protein sequence. Baci_subt: *B. subtilis* RA10 (Present work); Baci_amylo: *B. amyloliquefaciens* (NCBI Accession No. AFJ60402); Geob_stea: *Geobacillus stearothermophilus* (NCBI Accession No. EZP75177); Clos_buty: *Clostridium butyricum* (NCBI Accession No. KQB77061); Palu_yong: *Paludibacterium yongneupense* (NCBI Accession No. WP_028534323); Aero_salm: *Aeromonas salmonicida* (NCBI Accession No. EQC04707); Uncu_orga: uncultured organism (NCBI Accession No. AGD88872)

The cloned *Bgl*B gene was also sequenced, and deduced amino acid sequence was matched with the available GenBank database. The BLAST analysis showed highest identity (98%) with β-glucosidase from *Bacillus amyloliquefaciens* (NCBI Accession No. AFJ60402) belonging to the GH1 family protein. The multiple sequence alignment of the amino acid sequence of BglB with highly homologous GH1 enzymes was performed (Fig. 9). The sequence similarities showed the presence of conserved amino acid residues responsible for its catalytic activity. The catalytic acids (Asn^169^–Glu^170^), the catalytic nucleophile region E^377^NG and PROSITE motif PS00572 are well conserved in the BglB protein sequence which described the characteristic features of GH1 family protein.

**Fig. 10:**
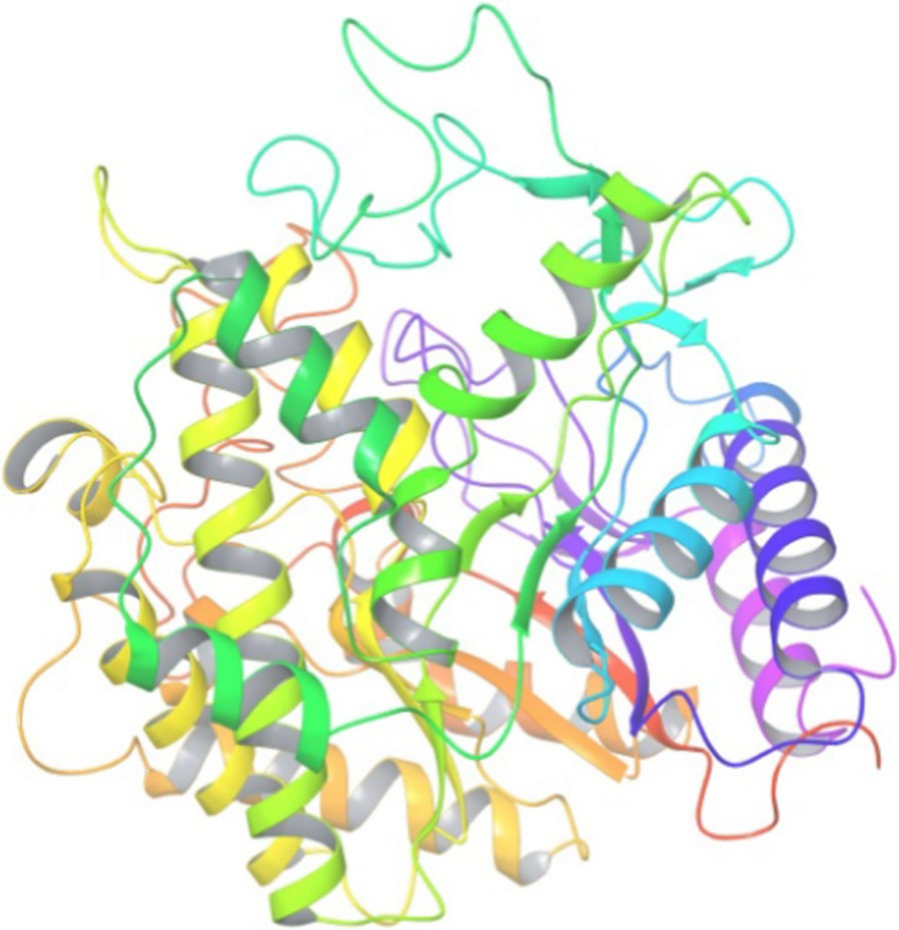
The predicted structural model of BglB from *B. subtilis* RA10 based on structure of β-glucosidase from *E. coli* (2XHY_A)

For 3D structure prediction, BglB sequence was modelled by SWISS-MODEL (http://swissmodel.expasy.org). Based on the similarity search in the protein database, 2XHY Chain A, crystal structure of β-glucosidase from *E. coli* was selected as the template for modelling (Fig. 10). The BglB sequence showed 40% of identity with the template sequence.

**Fig. 11:**
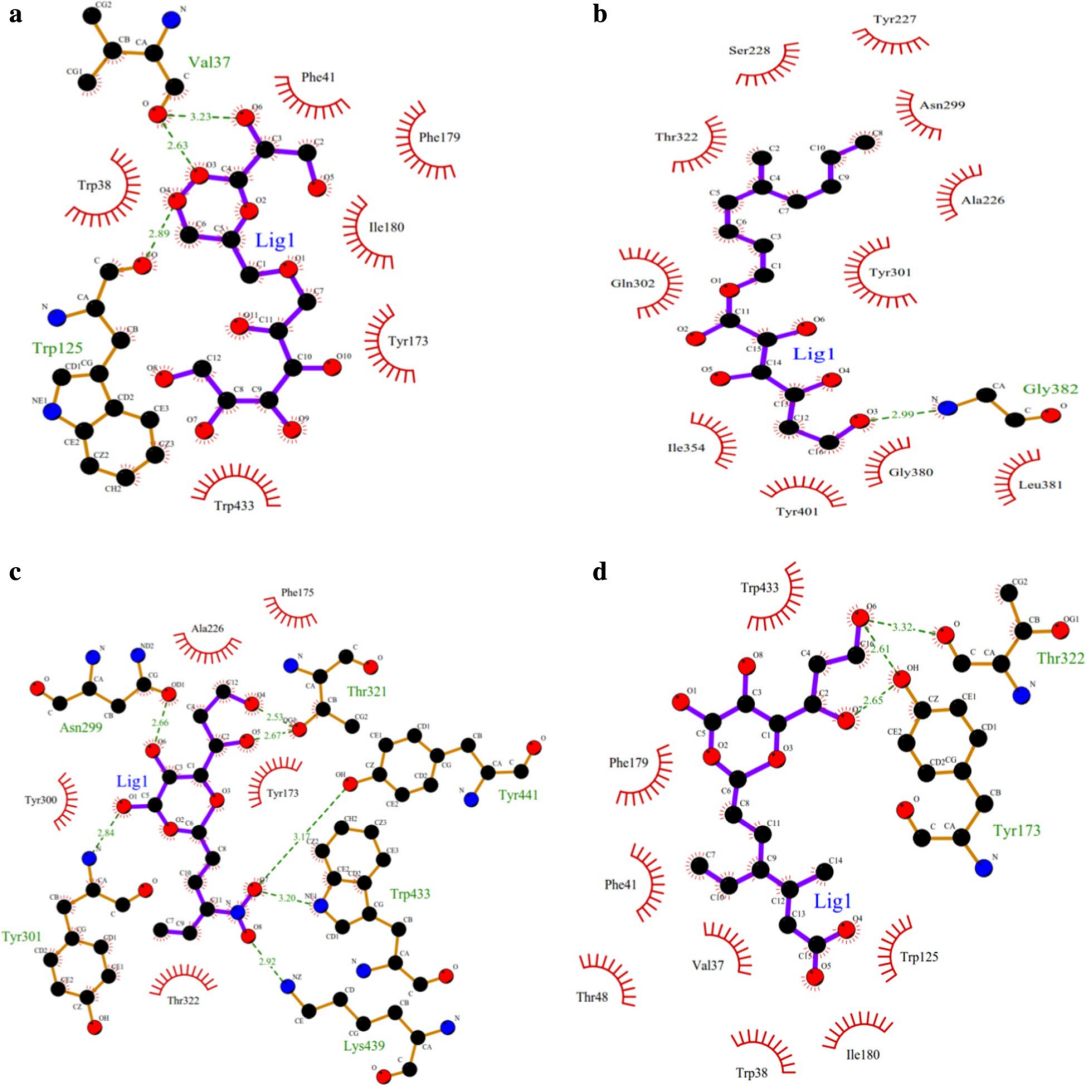
Substrate binding studies on the surface of β-glucosidase BglB protein. **a** binding of cellobiose with BglB; **b** binding of 6-bromo-2-naphthyl-β-d-glucopyranoside with BglB; **c** binding of 4-methylumbelliferyl-β-d-glucopyranoside with BglB; **d** binding of 4-nitrophenyl-β-d-glucopyranoside with BglB

The predicted model was further evaluated for docking studies to compare the binding efficiency of different substrates. Therefore, four different substrates including 4-methylumbelliferyl-β-d-glucopyranoside, 6-bromo-2-naphthyl-β-d-glucopyranoside, 4-nitrophenyl-β-d-glucopyranoside and cellobiose were docked against the predicted structural model (Fig. 11). Cellobiose was recorded as the least binding energy with − 7.03 kJ/mol followed by docking with 4-methylumbelliferyl-β-d-glucopyranoside with − 6.99 kJ/mol, least binding energy signifies the strong binding between substrate and enzyme. The inhibition constant for cellobiose substrate was also minimum (6.97 µM) as compared with other substrates. The amino acid residue Val37 formed hydrogen bonding with the cellobiose with bond length of 2.237 Å, and Trp38, Trp125, Phe41, Ile180, Trp433, Val37, Tyr173, Phe179 were recorded as the interacting residues contributing in the docking.

## Discussion

In the present investigation, the research was focused on the isolation of potent thermotolerant microorganisms capable of producing thermoactive β-glucosidase from compost and cow dung samples. For this concern, compost and cowdung samples were enriched in the presence of α-cellulose and/or cellobiose to isolate promising microbes with potentially high β-glucosidase production at 45 °C. The total viable count (TVC) of thermotolerant microbes was very low and ranged from 10^3^ to 10^5^ CFU (colony forming unit)/g of sample and showed the primary substrate based selection of microbial community. The molecular identification revealed the dominance of two groups including Actinobacteria and Firmicutes. The predominance of these genera in biomass compost and cow dung was previously reported by several researchers [12, 21–23].

The selected microbes were further explored for the quantitative estimation of β-glucosidase induced by different saccharides at 30–50 °C. All isolates were able to produce β-glucosidase enzyme at 30–50 °C, but higher enzyme production was observed at 40 °C. Isolate *B. subtilis* RA10 was able to produce maximum β-glucosidase enzyme among all eleven isolates. Several β-glucosidases were reported from thermophilic microbes like *Caldocellum saccharolyticum* [24], *Pyrococcus furiosus* [25], *Microbispora bispora* [26], *Thermoanaerobacter brockii* [27], and were reported to be producer of β-glucosidase enzyme at high temperature ranging from 40 to 90 °C.

For maximizing the β-glucosidase production by *B. subtilis* RA10, the optimization of medium components was performed using two statistical design including Taguchi experimental design and Box–Behnken design. The contribution and ranking of the factors and assigned levels revealed that the carbon source was the most important factor for stimulation on the β-glucosidase enzyme production by *B. subtilis* RA10. Paddy straw was previously used by researchers to induce the production of hydrolytic enzymes by different microorganisms [20, 28]. The pH 8 was optimum indicating alkaliphilic nature of the β-glucosidase enzyme.

The concentration of influential medium components was again optimized by Box–Behnken design. This result showed that high loading of carbon source inhibited β-glucosidase enzyme production by *B. subtilis* RA10. Similar results were obtained with beef extract—0.29% (w/v), PEG 6000—0.14% (v/v) and NaNO_3_—0.12% (w/v) where all the optimized values were lower than the highest concentration used in the design. This result showed the significance of optimization experiment where highest enzyme production was achieved by selecting minimal concentration of essential medium components and hence, more applicable for industrial purposes by reducing the cost of enzyme production. Both the statistical designs used in this study including Taguchi experiment and Box–Behnken design were the base for statistical validation of the data. The selection of media components and their concentration can also be analysed by one factor at a time (OFAT) method. However, a large number of experiments and subtle information about the factors interaction effect on enzyme production are the major bottlenecks of OFAT method. The Taguchi and Box–Behnken design help in revealing the statistical significant validation of interaction effect and factor contribution with almost accurate prediction for selected responses. These predictions can be easily conformed by the validation experiment. Therefore, these statistical tools are very commonly used in different bioprocesses.

The cellulase enzyme production is considerably influenced by the incubation temperature and may vary from different microbial strains [9, 20]. The growth and enzyme production within 30–60 °C proves the thermotolerant nature of β-glucosidase production from *B. subtilis* RA10. The activity was enhanced up to 40 °C and further decreased at 50 and 60 °C. *B. subtilis* RA10 belongs to the gram-positive bacteria with high GC content that facilitate the double-stranded structure of their DNA at high temperatures [29]. The existing hypothesis behind the occurrence of thermotolerant enzymes is based on the presence of reverse gyrase [30] or certain dinucleotides [31].

In order to regulate the complete utilization of the medium components as well as to increase β-glucosidase enzyme production, thermotolerant *B. subtilis* RA10 was cultivated under controlled optimized condition in a bioreactor with 5 L working volume. After 54 h, reducing sugar in the medium was consumed completely and simultaneously β-glucosidase enzyme production was enhanced. The result showed that monomeric sugars in the medium especially glucose inhibited the production of β-glucosidase [32]. In shake-flask experiment, the maximum β-glucosidase enzyme production (58.71 ± 1.73 IU/g) was achieved after 72 h of Incubation, whereas in stirred-tank bioreactor, the maximum β-glucosidase production (63.29 ± 1.73 IU/g) was obtained after 80 h of incubation which was 1.08-fold higher than the shake flask experiment. This enhancement of β-glucosidase production was due to controlled distribution of oxygen and pH into the growth medium throughout the experimentation in stirred tank bioreactor [33]. Subsequently, after achieving the maximum yield, β-glucosidase production was decreased in both the experiments including shake-flask and stirred-tank bioreactor. This might be due to the effect of inhibitors or protease production during the fermentation process [34].

The *B. subtilis* RA10 was grown under the optimized medium components and the obtained secretome was evaluated for saccharification of alkali-pretreated paddy straw at 50 °C. The experiment was performed to validate the effect of supplementation of β-glucosidase from *B. subtilis* RA10 with commercial cellulase (Celluclast^®^ 1.5L) for more efficient sugar yield. The saccharification result revealed that after supplementation of *B. subtilis* RA10 secretome, glucose yield was increased by 1.34-fold. This result confirmed that β-glucosidase from *B. subtilis* RA10 acts synergistically with commercial cellulase to serve better release of glucose from pretreated paddy straw. Similarly, many reports are available on the supplementation of β-glucosidase from external sources into commercial cellulase enzyme to improve the sugar release [35, 36]. In the present study, the glucose release was much higher as compared with previous reports [10, 37, 38]. Table 9 shows the comparison of different microbial β-glucosidase enzyme supplementations for improving the biomass hydrolysis.

**Table 9:** Comparison of different microbial β-glucosidase enzyme supplementations for improving the biomass hydrolysis

β-Glucosidase producing microbes	Biomass	Enzyme cocktail	Sugar release (mg/gds)	Enhanced saccharification (%) due to β-glucosidase supplementation	References
*Humicola grisea var. thermoidea*	Sugarcane bagasse	Cellulase of *T. reesei* RP-98	–	50	[36]
Microbial β-glucosidases from cow rumen metagenome	Corn stover	Celluclast, derived from *T. reesei*	195.67	20	[10]
		Cellulase supplemented with β-glucosidase	234.8		
β-Glucosidase gene from cow rumen	Barley straw	Cellulase from *Penicillium funiculosum*	–	150	[35]
*Penicillium piceum*	Corn stover	Cellulase from *T. reesei*; supplemented with β-glucosidase	–	15	[38]
*Pseudomonas lute a*BG8	Paddy straw	Celluclast^®^ 1.5L	385.28	42.33	[48]
		Celluclast^®^ 1.5L supplemented with β-glucosidase from *P. lutea* BG8	548.4		
*Bacillus subtilis* RA10	Paddy straw	Celluclast^®^ 1.5L	437.79	53.63	Present work
		Celluclast^®^ 1.5L supplemented with β-glucosidase from *P. lutea* BG8	582.58		

Furthermore, the full length gene of GH1 family β-glucosidase was amplified from *B. subtilis* RA10 and cloned into the pET28a^+^ vector for overexpression. Only few GH1 family β-glucosidase enzymes were overexpressed in the heterologous expression system from different microorganisms like *Acidilobus saccharovorans* [39], *Pyrococcus horikoshii* [40], *Bacillus* sp. [41] and *Streptomyces* sp. [42]. The enzyme characterization of purified protein revealed high β-glucosidase activity even at high temperature and broad range of pH. Most of the β-glucosidase enzyme from *Bacillus* origin including *B. halodurans* [43], *B. subtilis* [44], *Bacillus* sp. [41] and *Anoxybacillus* sp. [45] revealed efficient activity within temperature range of 40–55 °C at slightly acidic condition. Hence, the results obtained are unique since overexpressed β-glucosidase enzyme from *B. subtilis* RA10 maintained its activity at high temperature and broad range of pH. The β-glucosidase enzyme from *B. subtilis* RA10 is thermostable in nature and can be further recycled for the saccharification purpose. According to the Zhang et al. [46], 100–200 g of cellulases is consumed for one gallon of bioethanol production. This high consumption of cellulase enzyme increases the production cost of whole bioethanol process. The involvement of thermostable cellulases may improve the efficiency of the hydrolysis production process. Although cellulases have substrate binding sites which limit the recovery of enzyme after the saccharification, the rate-limiting enzyme of saccharification, β-glucosidase enzymes do not have any such substrate binding module and hence cannot be adsorbed by the cellulose content [47]. This property of β-glucosidase enzyme can be utilized to recover this enzyme after saccharification if it retains high activity after the hydrolysis step.

The substrate specificity of overexpressed β-glucosidase enzyme from *B. subtilis* RA10 was analysed by in silico method. The docking studies revealed that cellobiose was recorded as the least binding energy with − 7.03 kJ/mol and signified strong binding between substrate and enzyme. The variation in the binding efficiency of modelled protein with different substrates is important for further studies. This information may infer new insights to develop engineered protein for improvising the enzyme activity at broad range of temperature and pH so that the enzyme will be more applicable for different industrial purposes.

## Conclusions

In the present study, thermotolerant microbial isolates with β-glucosidase enzyme characterization and their genetic information gathered from two environmental niches have strengthened the knowledge about β-glucosidase enzyme. The microbial resource is having potential to produce high titer of β-glucosidase enzyme with novel and unique characteristics. β-glucosidase enzymes produced by *B. subtilis* RA10 have shown further improvement in hydrolytic efficiency of biomass which will play a pivotal role in the biorefinery industries. In a nutshell, enzymatic characterization, 3D structure prediction, substrate-based docking studies and hydrolytic potential are sufficient to explain the novelty of thermostable β-glucosidase from *B. subtilis* RA10.

## Additional file

**Additional file 1: Figure S1.** Qualitative screening of β-glucosidase producing microbes on agar medium containing esculin with or without glucose. Thermotolerant isolates grown on esculin [A] and esculin with glucose [B]. **Figure S2.** PCR amplification of *Bgl*B gene and 1437 bp fragment from nuclear DNA of *B. subtilis* RA10. Lane M, Lambda DNA/*Eco*RI + *Hind*III Marker; lane 1, amplified PCR product. **Figure S3.** Determination of *V*_max_ and *K*_m_ for purified β-glucosidase enzyme.

## Abbreviations

RMM: Reese’s minimal medium
RSM: response surface methodology
CMC: carboxymethyl cellulose
mM: millimolar
DNA: deoxyribonucleic acid
rRNA: ribosomal ribonucleic acid
PCR: polymerase chain reaction
3D: three-dimensional
ANOVA: analysis of variance
TVC: total viable count
GH1: glycosyl hydrolase family 1
