# Supplementary material for: Bioprospecting of novel thermostable β-glucosidase from Bacillus subtilis RA10 and its application in biomass hydrolysis
Source: Biotechnol Biofuels. 2017 Oct 30;10:246. doi: 10.1186/s13068-017-0932-8 (PMC5663093; doi:10.1186/s13068-017-0932-8)
Supplement: Supplementary file 1 — Additional file 1: Figure S1. Qualitative screening of β-glucosidase producing microbes on agar medium containing esculin with or without glucose. Thermotolerant isolates grown on esculin [A] and esculin with glucose [B]. Figure S2. PCR amplification of BglB gene and 1437 bp fragment from nuclear DNA of B. subtilis RA10. Lane M, Lambda DNA/EcoRI + HindIII Marker; lane 1, amplified PCR product. Figure S3. Determination of V max and K m for purified β-glucosidase enzyme. [file 13068_2017_932_MOESM1_ESM.docx]

**Figure S1:** Qualitative screening of β-glucosidase producing bacterial isolates on agar medium containing esculin [A] and esculin with glucose [B].


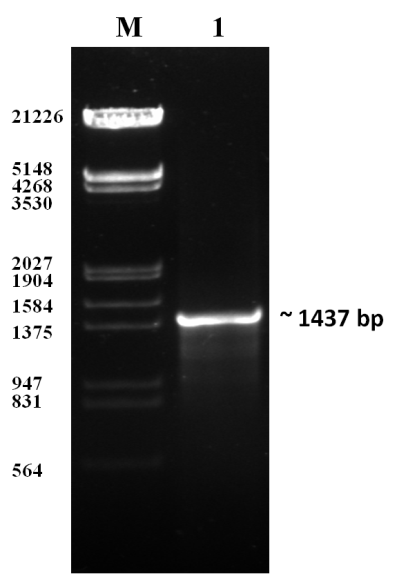


**Figure S2:** PCR amplification of *Bgl*B gene and 1437 bp fragment from nuclear DNA of *B. subtilis* RA10. lane M, Lambda DNA/*Eco*RI+*Hind*III Marker; lane 1, amplified PCR product.


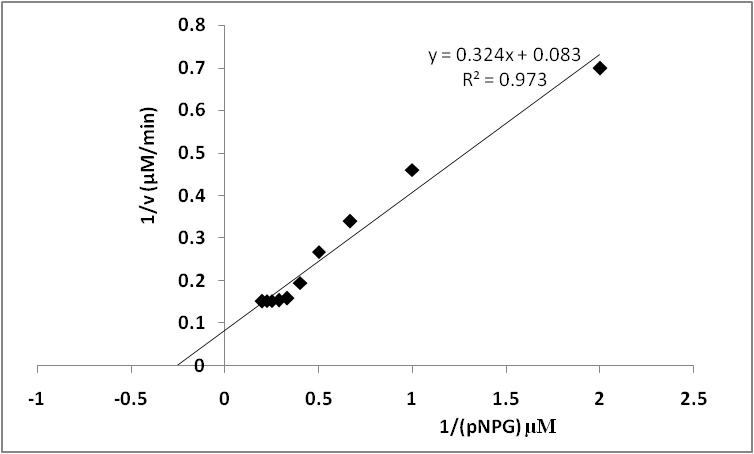


**Figure S3: Determination** of V_max_ and K_m_ for purified β-glucosidase enzyme.
